# Integration of Different Graphene Nanostructures with PDMS to Form Wearable Sensors

**DOI:** 10.3390/nano12060950

**Published:** 2022-03-14

**Authors:** Shan He, Yang Zhang, Jingrong Gao, Anindya Nag, Abdul Rahaman

**Affiliations:** 1 School of Chemistry and Chemical Engineering, Guangzhou University, Guangzhou 510006, China; he0091@gmail.com (S.H.); 2112005093@e.gzhu.edu.cn (Y.Z.); 2 Institute for NanoScale Science and Technology, College of Science and Engineering, Flinders University, Bedford Park 5042, Australia; 3 School of Food Science and Engineering, South China University of Technology, Guangzhou 510640, China; 4 Faculty of Electrical and Computer Engineering, Technische Universität Dresden, 01062 Dresden, Germany; 5 Centre for Tactile Internet with Human-in-the-Loop (CeTI), Technische Universität Dresden, 01069 Dresden, Germany

**Keywords:** graphene, wearable, sensors, reduced graphene oxide, nanoplatelets

## Abstract

This paper presents a substantial review of the fabrication and implementation of graphene-PDMS-based composites for wearable sensing applications. Graphene is a pivotal nanomaterial which is increasingly being used to develop multifunctional sensors due to their enhanced electrical, mechanical, and thermal characteristics. It has been able to generate devices with excellent performances in terms of sensitivity and longevity. Among the polymers, polydimethylsiloxane (PDMS) has been one of the most common ones that has been used in biomedical applications. Certain attributes, such as biocompatibility and the hydrophobic nature of PDMS, have led the researchers to conjugate it in graphene sensors as substrates or a polymer matrix. The use of these graphene/PDMS-based sensors for wearable sensing applications has been highlighted here. Different kinds of electrochemical and strain-sensing applications have been carried out to detect the physiological signals and parameters of the human body. These prototypes have been classified based on the physical nature of graphene used to formulate the sensors. Finally, the current challenges and future perspectives of these graphene/PDMS-based wearable sensors are explained in the final part of the paper.

## 1. Introduction

The end of the 20th century has seen burgeoning growth in the field of nanotechnology-based devices. Different kinds of nanomaterials [1,2] have been used to devise sensors for domestic and industrial applications. In earlier times, when the large integrated circuits were used for sensing applications, their impact in terms of sensitivity and longevity was largely dependent on their immediate environment. As a replacement, the researchers have started fabricating small-scaled sensors [3,4], which not only have enhanced characteristics but can also be deployed for additional applications. The initial growth of these sensors took place as semiconducting prototypes [5,6,7], where the substrates and electrodes were formed using silicon and metallic nanoparticles such as gold, chromium, and platinum [8,9], respectively. These silicon sensors were formed using microelectromechanical system (MEMS) techniques that consisted of techniques such as conventional photolithography [10,11] to form the designated electrodes. Although these sensors were used for different environmental [12,13] and gas-sensing [14,15] applications, some disadvantages were associated with their fabrication cost. One of the primary disadvantages is that the development of these sensors requires very expensive cleanrooms and foundry facilities, thus increasing the assembly cost even for a few sensors [16,17]. This caused the researchers to further develop sensors that included flexible materials. The sensors have been devised using extensive printing techniques [18,19] such as screen printing [20,21], inkjet printing [22,23], 3D printing [24,25], offset lithography [26,27], flexography [28,29], and gravure printing [30,31].

The fabrication techniques have been employed to process certain polymers and nanomaterials to form prototypes. A wide range of polymers and nanomaterials have been processed to develop flexible sensors [32,33], which have been selected based on the applications of the prototypes. Regarding the polymers, some of the common ones include polydimethylsiloxane (PDMS) [34,35,36], polyethylene terephthalate (PET) [37,38,39], polyimide (PI) [40,41,42], and polyethylene naphthalate (PEN) [43,44,45]. These polymers have been either used to form the substrates of the sensors or as the polymer matrix in the nanocomposites, where the nano-fillers have been added at defined proportions. In recent times, conductive polymers have been used to lower the manufacturing cost of the thin films and minimize the limitations of the nanocomposite-based electrodes. Some of the common conductive polymers used to form the flexible sensors are PEDOT: PSS [46,47,48], polyaniline (PANI) [37,49,50], polyacetylene (PA) [51,52,53], and polypyrrole (PPy) [54,55,56]. Their conjugation assists in inducing additional functional groups that add material properties to the prototypes. The biocompatible polymers are highly advantageous due to certain attributes such as renewability, biodegradability, and cost-effectiveness. These polymers have been largely preferred to devise wearable sensors that have been used to detect chronic and acute anomalies. The prototypes have been used externally by attaching them to the body and internally by implanting them inside the human body. Among the biocompatible polymers, PDMS is the most common one due to its chemically inert nature, high permeability for gases, good thermal stability, optically transparency, hydrophobic nature, and its excellent interfacial bonding with nanofillers in the composites.

**Figure 1:**
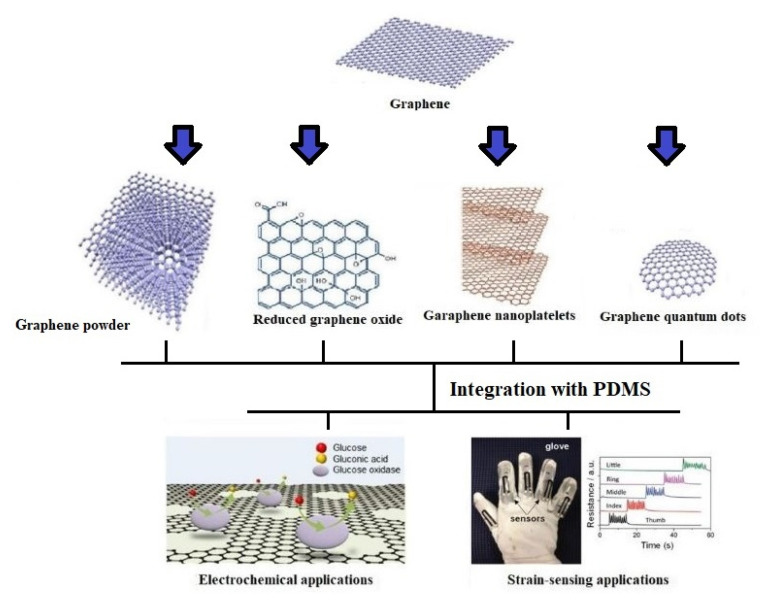
Use of different kinds of graphene nanomaterials for electrochemical [104] and strain-sensing [105] applications by integrating them with PDMS. Reproduced from Kwon, S.S., Shin, J.H., Choi, J., Nam, S., and Park, W.I., 2017. “Defect-mediated molecular interaction and charge transfer in graphene mesh–glucose sensors”. *ACS Applied Materials & Interfaces*, *9*(16), pp. 14216–14221; “A graphene-based electrochemical device with thermoresponsive microneedles for diabetes monitoring and therapy”. *Nature nanotechnology*, *11*(6), pp. 566–572; and Irani, F.S., Shafaghi, A.H., Tasdelen, M.C., Delipinar, T., Kaya, C.E., Yapici, G.G., and Yapici, M.K., 2022. “Graphene as a Piezoresistive Material in Strain Sensing Applications”. *Micromachines*, *13*(1), p. 119.

Similar to the polymers, different nanomaterials have been considered to develop flexible sensors. These nanomaterials can be distinguished into two classes, including carbon-based allotropes [57,58,59] and metallic nanomaterials [60,61,62]. While materials such ascarbon nanotubes (CNTs) [63,64,65], graphene [66,67,68], and graphite [69,70,71] fall in the former category, the second class consists of different kinds of physiochemical structures such as nanoparticles [72,73,74], nanowires [75,76,77], nano-beads [78,79,80], nano-ribbons [81,82,83], and quantum dots [84,85,86]. Regarding the development of wearable sensors, carbon-based allotropes have been preferred over the other allotropes due to their high biocompatibility and excellent electromechanical characteristics. Among the carbon-based allotropes, graphene [87,88,89] has been one of the forerunning materials due to its ultralight nature, high tensile strength, high charge carrier mobility, and high heat conduction capability. It has been used for a wide range of biomedical [90,91,92], industrial [93,94,95], and environmental [96,97,98] applications. Wearable sensors have efficiently monitored the human body’s physiological signals [99,100] and motions [101,102]. Certain attributes of the wearable sensors such as high accuracy, reliability, and repeatability of the responses have led the researchers to use them to analyze and provide healthcare-related data to the monitoring unit [101,103]. Signals from critical organs have been detected in patients for rehabilitation to improve their quality of life. Figure 1 [104,105] represents certain kinds of graphene nanomaterials that are used along with PDMS for electrochemical and strain-sensing applications. Even though a lot of topical reviews have been written on the use of graphene [106,107] and PDMS-based [108,109] sensors, the illustration of the conjugated use of these two materials in terms of fabrication and implementation has not been done yet. A significant elucidation of the electromechanical effect of the graphene/PDMS-based sensors for wearable sensing applications has not been done yet. This paper highlights the use of graphene/PDMS-based sensors for different kinds of wearable sensing applications. Categorization of the physical structures of graphene has been done to display their effect alongside PDMS as wearable sensors.

The paper has been organized as follows. The brief introduction regarding the significance of graphene, PDMS, and wearable sensors given in Section 1. Section 2 highlights a classification of different types of graphene/PDMS-based sensors. It explains the nature of the processed materials used to form the prototypes and their subsequent applications as wearable sensors. The sensors have been categorized into four types, each differing from the ones in terms of the physicomechanical structure of graphene. The four categories of graphene are nanopowder [110,111], reduced graphene oxide (rGO) [112,113], nanoplatelets [114,115], and quantum dots [116,117]. Then, the challenges related to the current graphene/PDMS-based sensors have been highlighted, alongside some of their possible remedies. The conclusion is drawn in Section 4.

## 2. Graphene/PDMS-Based Sensors

The design and development of wearable sensors using biocompatible materials have been done for quite a few years. These biodegradable prototypes have been formed using biomaterials that increase the biocompatibility between the sensors and the human body. The design of the device structure has been done in a way to increase the long-term stability in their responses during the monitoring of physiological signals. Some of the other advantages provided by these biocompatible sensors are the inclusion of low-cost devices for multifunctional applications, the ability to perform in vitro and in vivo experiments, high accuracy, reduced toxicity, and minimal side-effects during and following the experiments [118]. The high sensitivity and fast response time of the wearable sensors have led researchers to use them with activities related to daily life. When used as reliable diagnostic devices, these prototypes have been able to provide a linear response for wearable electrochemical [119] and strain-sensing [120] applications. Graphene, having unique electronic and chemical properties, can detect micro-movements [121] and operate as biomarkers to different kinds of ions [122]. Their high sensitivity towards molecules and mechanical bending have allowed the sensors to detect changes in minuscule concentrations. In order to analyze the performances of graphene sensors with respect to other nanomaterials, comparative studies have been depicted in Table 1 and Table 2. The comparison has been made on the basic parameters used to determine the qualities of electrochemical and strain sensors. As a result of huge spectrum of electrochemical sensing applications, the ones related to glucose sensing has been included here. It is seen that the performances of these graphene-based prototypes excel the ones developed using other nanomaterials. The reasons behind this can be attributed to the enhanced characteristics of graphene, PDMS, and other associated processed materials. An optimization process was carried out for each prototype on the number of raw materials used to form them. The following sub-sections showcase some of the significant examples of the use of wearable sensors that are formed using graphene and PDMS. The fabrication process and their respective applications for each of the prototypes are briefly explained.

### 2.1. Graphene Nanopowder-Based Devices

**Figure 2:**
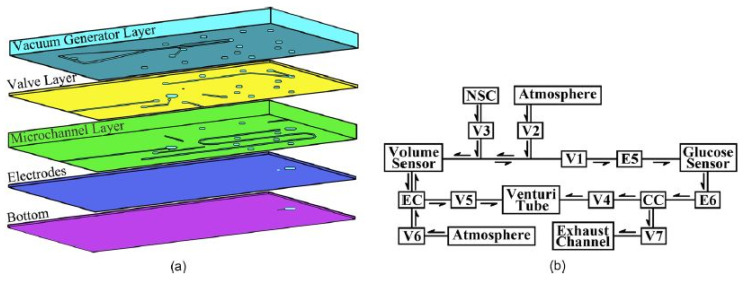
(**a**) Different layers of the chip. (**b**) Working process of the devices [123]. Reproduced from Pu, Z., Zou, C., Wang, R., Lai, X., Yu, H., Xu, K. and Li, D., 2016. “A continuous glucose monitoring device by graphene modified electrochemical sensor in microfluidic system”. *Biomicrofluidics*, *10*(1), p. 011910.

The formation of electrochemical sensors using graphene has been very advantageous due to their excellent ion carrier capabilities. Due to the huge mass of applications of electrochemical sensors, the authors have restricted the discussion to glucose-based electrochemical sensors to ease the realization of the fabrication of sensors and their working mechanism for the readers. The research done by Parmeggiani et al. [42] shows the formation of multifunctional laser-induced graphene sensors using both PDMS and PI as elastomeric substrates. Novel polymeric composites were formed, where PI was dispersed inside the PDMS matrix to obtain the resultant templates for the laser-induction process. A simple casting process was performed on the silicone elastomer to generate flexible electrodes that can suffice to adapt to different kinds of shapes and surfaces. The mixing of the PDMS and PI polymers was done at a ratio of 1:1 for 10 min, followed by degassing and curing them at a temperature of 60 °C for an hour. The laser writing process was carried out with optimized power value, frequency, and duty cycle of 30 W, 5 kHz, and 100%, respectively. The testing of the sensors was done using the cyclic voltammetry (CV) technique to determine the changes in current density with respect to time. The testing of the sensors was done at a temperature of 70 °C for characterizing and testing them. The rise in the device capacitance was around 60% with respect to the bending radius for the chosen rectangular shape. One of the interesting works related to the fabrication of single-layer graphene film/PDMS-based wearable sensors and implementing them for electrochemical sensing applications can be seen in [123]. Microfluidic systems were formed for continuous monitoring of glucose molecules. Three-electrode sensors were formed and embedded into a microfluidic chip for sensing applications without interference from foreign body reactions. The prototypes consisted of five different PDMS layers, which were processed using micromolding techniques. Figure 2a,b [123] shows the schematic diagram of the different layers and the working mechanism of the devices. The five layers consisted of a vacuum generator layer, a valve layer, a microchannel layer, an electrode layer, and a bottom layer. Graphene and gold nanoparticles were used to develop the working electrodes of these sensors. Electrodeposition of the gold nanoparticles was done on the graphene layer to enhance the overall electron transfer rate and overall sensitivity. The three-electrode systems consisting of working, counter, and reference electrodes were formed on glass substrates. While the reference electrodes were formed by sputtering chromium and platinum layers, the graphene composites were used as working electrodes. The electrochemical polymerization of graphene was done on the surface of the working electrodes for the detection of glucose molecules. The prototypes were employed for the measurements of low glucose levels, and they were able to detect hypoglycemia with excellent performances. The linear range and limit of detection (LOD) of 10–1620 ppm and 10.44 ppm (0.44 mg/dL), respectively.

Wang et al. [139] showed the use of super-elastic graphene ripples for the development of flexible strain sensors. A buckling approach of graphene powder and graphene ribbons was used to form the resultant stretchable elastomeric substrates.

The mechanical cleavage method was used to form the graphene samples, followed by using a lithography technique to pattern the silicon dioxide (SiO_2_) substrates. Then, the samples were treated with certain techniques such as spin-coating, oxygen plasma etching, and e-beam lithography techniques. The graphene was transferred on the PDMS films which were formulated over a wide range of pre-strains. The prototypes operated on the nanoscale periodical buckling process subsequent to releasing of strain. This was followed by performing metal deposition of titanium and gold with thicknesses of 1.5 nm and 30 nm, respectively. Finally, these graphene ripples were transferred on PDMS substrates, along with the removal of the polymethyl methacrylate (PMMA) layer to form the final sensors. The shape of these ripples changed with respect to the pre-strain of the original shape and substrates. These strain sensors were operated as novel flexible electronic devices.

Another example related to the fabrication and implementation of graphene-based strain sensors can be seen in the work done by Yang et al. [131]. The sensors consisted of composite films that consisted of layers formed using Ti_3_C_2_T_x_, graphene, and PDMS as processed materials. The working principle of these sensors was based on two layers that included a flexible graphene/PDMS nanocomposite and a Ti_3_C_2_T_x_ dominated brittle layer. A synergy was maintained between the top and bottom to obtain a high and steady response during strain-sensing applications. An anode coverage electrochemical process was used to synthesize graphene particles. The technique involved the use of graphene and platinum foils with defined dimensional specifications. The intermediate results were purified in water and dried at a temperature of 50 °C to obtain the conductive nanomaterial. The composite films consisting of Ti_3_C_2_T_x_ and graphene were formed via developing suspensions with different weight percentages of each of these nano-elements. Finally, the strain sensors were fabricated by initially pre-polymerizing PDMS on molds having sizes of 80 × 15 × 1 mm^3^. Then, the conductive thin stripes were transferred to the PDMS substrates and cured at a temperature of 50 °C for an hour. The prototypes had a gauge factor (G.F.) of 190.8 and 1148.2 for low and high strain ranges of 0–56% and 52.6–74.1%, respectively. The sensors also displayed high cycling stability for over 5000 cycles, in addition to high linearity of R^2^ > 0.98 and a LOD of −0.025%.

### 2.2. Reduced Graphene Oxide-Based Devices

**Figure 3:**
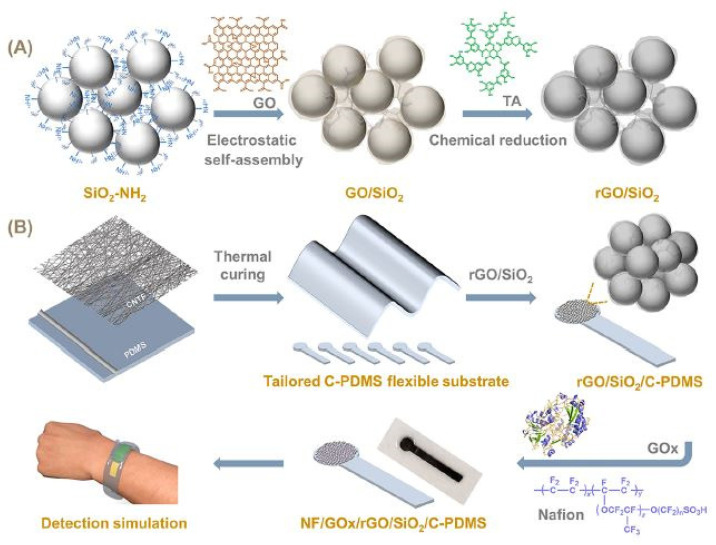
Illustration of the fabrication steps of (**A**) electroactive rGO-based nanocomposites and (**B**) flexible glucose biosensors [124]. Reproduced from Xu, M., Zhu, Y., Gao, S., Zhang, Z., Gu, Y. and Liu, X., 2021. “Reduced graphene oxide-coated silica nanospheres as flexible enzymatic biosensors for detection of glucose in sweat”. *ACS Applied Nano Materials*, *4*(11), pp. 12442–12452.

The flexible wearable sensors developed using rGO and PDMS have been very efficient due to their high mechanical strength, molecular barrier abilities, and high electrical conductivity. These materials can provide high stability, durability, and repeatability of the responses. The high surface area of rGO is advantageous for electrochemical applications as they assist in the adsorption of the target molecules. Xu et al. [124] depicted the fabrication of flexible enzymatic biosensors to detect glucose molecules in sweat. The 3D prototypes were formed using nanocomposites that consisted of rGO-coated silica nanospheres. Figure 3 [124] illustrates the schematic diagram of the fabrication steps of rGO-based nanocomposites and flexible glucose biosensors. After preparing GO using the modified Hummers method, the final samples were dialyzed for a week and frozen to obtain the solid GO powder.

The nanocomposites were formed by assembling rGO nanosheets on the surface of SiO_2_ nanospheres using two separate processes. Initially, the electrostatic interaction and in situ reductions by tannic acid techniques were employed, followed by the dropping of the rGO/SiO_2_ nanocomposites, glucose oxide, and Nafion solution on the conductive substrates to form the resultant sensing area of the prototypes. The conductive substrates were formed using CNTs film and PDMS. After the pre-polymer and curing agents of PDMS were mixed, they were coated on plexiglass substrates using a 60 µm coating wire rod. The electrical conductivity of these PDMS substrates was increased by coating, pressing, and curing of the CNTs film on them at 80 °C for four hours. The specific area of these biosensors with increased sensing and recognition sites exhibited excellent dynamic characteristics such as a good linear detection range of 0.1–9 mM. The sensitivity and LOD of the sensors were 60.8 µA·mM^−1^·cm^−2^ and 3.7 µM, respectively.

Another example of the use of rGO/PDMS-based wearable sensors for electrochemical sensing applications can be shown in work done by Moon et al. [140]. Stretchable, room-temperature operable, chemiresistive gas sensors were formed by using nanohybrids comprising of rGO and vertically grown zinc oxide (ZnO) nanorods. These nanorods were grown on stress-absorbable, elastic 3D micropatterned PDMS substrates. The fabrication process was carried out by depositing thin alumina substrate on the PDMS substrates. These alumina layers were modified using different chemicals to increase the adsorption capability of GO nanosheets. Then, GO solutions were deposited on the modified alumina substrates to form the sensing area of the prototypes. Finally, interdigitated electrode designs were formed on which chromium/gold metals were deposited using e-beam and thermal evaporation processes. The thickness of the chromium and gold layers were 7 nm and 63 nm, respectively. Then ZnO nanorods were deposited on the GO to enhance their adsorption capability, followed by functionalizing the former nanomaterial using (3-aminopropyl) triethoxysilane (APTES)-based aqueous solutions. The deposition of ZnO nanorods on the substrates was done using the hydrothermal method, after which, the samples were sealed with Teflon tape and heated at 85 °C for 17 h. The gas sensors were used at room temperature to detect low concentrations of nitrogen dioxide gas. Other than certain attributes such as high sensitivity, and rapid response and recovery times, the prototypes were able to detect the gas with a LOD of 40 ppb.

**Figure 4:**
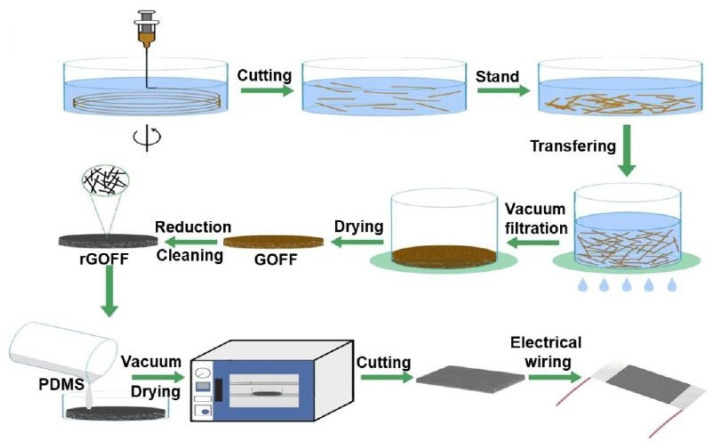
Schematic diagram of the fabrication of the rGOFF/PDMS-based pressure sensors [132]. Reproduced from Jiang, X., Ren, Z., Fu, Y., Liu, Y., Zou, R., Ji, G., Ning, H., Li, Y., Wen, J., Qi, H.J., and Xu, C., 2019. “Highly compressible and sensitive pressure sensor under large strain based on 3D porous reduced graphene oxide fiber fabrics in wide compression strains”. *ACS Applied Materials & Interfaces*, *11*(40), pp. 37051–37059.

Apart from the electrochemical sensing applications, the rGO/PDMS-based sensors have also been used for strain sensing applications. One of the interesting works can be shown in the research done by Jiang et al. [132], where highly compressible and sensitive pressure sensors were developed using 3D porous rGO fiber fabrics (rGOFF). Figure 4 [132] illustrates the fabrication of these rGO/PDMS-based pressure sensors. After synthesizing GO using the improved Hummers method, these nanomaterials were chemically treated to form chemically expanded graphite. Then, the samples were incubated at a temperature of 35 °C for four hours. The obtained GO fibers having a length of around 4–6 mm were treated with vacuum filtration method and were dried at 60 °C for 3 h. The GO fabrics were then reduced to form rGOFF using hydrazine hydrate vapors at 95 °C for 12 h. The rGOFF was finally placed on the petri dishes on which PDMS was poured, degassed, and cured to form the composite-based prototypes. The samples were then cut into specific sizes and connected to aluminum foil via silver conducting resins. The final step involved drying them at 60 °C for 3 h to solidify the glue before using them for characterization and experimental purposes. The interfused fiber-to-fiber interfaces assisted the sensors in generating efficient performances. While the sensors were able to respond to a wide range of strain ranging between 0.24% and 70%, the G.F., LOD, and response time were 1668.48, 1.17 Pa, and 30 milliseconds, respectively. The change in response of the prototypes was detected in terms of relative resistance to detect the mechanical changes happening due to loadings and compressions.

One of the interesting works highlighting the development of highly stretchable and ultrasensitive wearable strain sensors using rGO and PDMS processing materials can be shown in [141]. The prototypes were formed using template-induced assembly and polymer coating processes. Hydrothermal assisted synchronous reduction and assembly of GO sheets over copper mesh was initially made after preparing the GO using the modified Hummers method. Then, selective coating of PDMS over copper mesh/rGO was done by dividing the latter into three areas via the etching technique. Then, the samples were vacuum dried at 120 °C for 2.5 h for polymerization reaction of the PDMS monomers on the rGO/copper mesh. Further selective etching of the copper mesh was done by treating them chemically for 12 h, then washing them with deionized (DI) water and drying them in the air. The chemical treatment assisted in oxidizing the copper ions with the help of iron ions. Some of the attributes of the sensors were long-term durability and high selectivity for various disturbances. The sensors had a high G.F. of 630 under 21.3% applied strain. The sensors also showed high selectivity for bending and twisting angles of 180° and 90°, respectively, and external temperature ranging between 30 and 63.5 °C.

### 2.3. Graphene Nanoplatelets-Based Devices

After the graphene and oxide form, graphene nanoplatelets (GNPs) have been the third most common form that has been widely used to integrate with PDMS polymer for forming wearable sensors. The size of the particles was less than a micron and each one had a density between 0.2 and 0.4 g/cm^3^. Wiederoder et al. [142] developed chemiresistive sensors using polymers and GNPs to detect gas at specified concentrations. GNPs were mixed with polycaprolactone (PCL) via airbrush deposition technique, followed by optimizing the parameters for sensing performance. The ratio of GNP and PCL was optimized between 3 and 21 wt %, where the maximum sensing performance and signal-to-noise (SNR) ratio were obtained at 15 wt % and 18 wt %, respectively. The sensors were formed using a PDMS mask that had a diameter of 6 mm. The masking of the electrodes was done using PDMS, followed by placing them below an immobilized airbrush on a hotplate. The temperature of the hotplate was adjusted to 30 °C. The final steps included dispensing the GNP/PCL mixture on the substrates using short-duration sprays. The volume used for coating purposes was fixed between 50 and 250 µL. Some of the advantages of the sensors are the high robustness and repeatability of the responses. The detection of ethanol vapors was carried out in terms of relative resistance with respect to deposited GNP/PCL volume. With the mixtures being developed with a concentration of 18 wt % GNP, an enhanced response in terms of output and SNR were obtained at deposited amounts of 150 µL and 200 µL, respectively. Another interesting example showcasing the use of GNP for electrochemical sensing applications can be seen in [143]. Polymer electrolyte membrane fuel cell catalysts were developed using composites that consisted of platinum nanoparticles (Pt NPs), GNPs, and silicone rubber as processing materials. Two different types of GNPs having surface areas of 181 m^2^/g and 745 m^2^/g were used with and without Pt NPs. Initially, pyrolysis of GNPs was done by mixing them with melamine at a 1:1 molar ratio. Then, the samples were heated for a specified time, followed by cooling them naturally. The microwave irradiation technique was used to load these supportive Pt NPs. It was carried out at a power of 800 W for a min. Finally, the solutions were cooled down again and centrifuged at a speed of 7000 RPM for 15 min. The last step included drying the samples at a temperature of 100 °C in the drying oven. The electrochemical activity of these GNPs/Pt NPs was carried out on various catalyst binders, including PDMS and conventional Nafion solution and PDMS. The performance of the PDMS-based sensors was better than the Nafion one at a particular voltage of 0.6 V. The current densities of the two varied substrates were 531.8 mA/cm^2^ and 437 mA/cm^2^, respectively.

**Figure 5:**
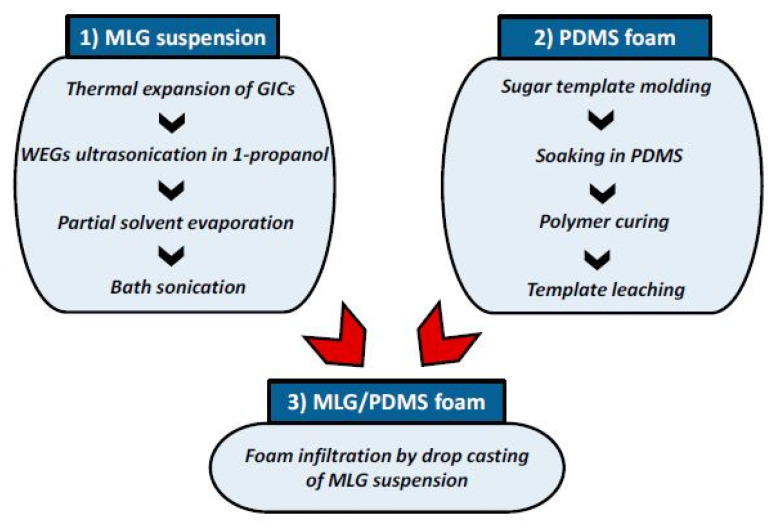
Illustration of the flow of fabrication steps of piezoresistive multilayered GNP/PDMS foams [144]. Reproduced from Rinaldi, A., Tamburrano, A., Fortunato, M. and Sarto, M.S., 2016. “A flexible and highly sensitive pressure sensor based on a PDMS foam coated with graphene nanoplatelets”. *Sensors*, *16*(12), p. 2148.

Apart from electrochemical sensing applications, the GNP/PDMS-based sensors have also been used as strain sensors. For example, Rinaldi et al. [144] developed flexible and highly flexible sensitive pressure sensors based on PDMS foam-coated with GNPs. Some of the advantages of these multilayered structures include low cost, softness, and high mechanical flexibility. Figure 5 [144] shows the fabrication steps of these sensors. Initially, the formation of colloidal suspensions of multilayered GNPs was done using the thermal expansion process. It included using a muffle furnace at 1150 °C for 5 s. The formed agglomerated nanofillers were re-dispersed to form stable precipitation by sonicating them for 30 min. This was followed by forming PDMS foams and infiltrating them with the multilayered GNPs. A direct template technique was used to form the PDMS foams. In order to induce solvent evaporation, the infiltration process was done inside an environmental chamber at 110 °C to induce solvent evaporation. Several cycles were carried out to infiltrate 0.25 mL of suspension after optimizing the multilayered GNPs with respect to the PDMS foams. While these prototypes have been used as piezoresistive pressure sensors, various experiments were conducted to detect the responses of the sensors with respect to different loads. The sensitivity was 0.23 kPa^−1^ for a corresponding applied pressure of 70 kPa. The prototypes detected compressive stress of 10 kPa and a pressure variation of around 1 Pa.

Another interesting example related to the use of GNPs/PDMS-based wearable sensors for healthcare applications can be seen in the work done by Baloda et al. [133]. A few of the attributes of these flexible resistive strain sensors are excellent sensitivity and high stretchability. An optimization process was carried out to determine the weight percentage of GNP to be mixed with PDMS substrates. After a value of 40 wt % was chosen, the manual printing process was carried out to directly coat the substrates via paintbrush. The samples were then cured at a temperature of 90 °C for 90 min. Apart from PDMS, some of the other nanomaterials with which GNPs were successfully integrated were PET, PI, and carbon fiber. The prototypes were operated to detect human motions such as wrist pulse measurement, finger bending movement, and flexible pipes. The sensors were also tested to detect their capability of detecting arterial blood pressure and as wearable diagnostic electronic devices. When stretched up to 65%, the sensors showed a G.F. of 62.5.

### 2.4. Graphene Quantum Dots-Based Devices

Apart from the above-mentioned graphene physiochemical structures, another type of graphene that has also been integrated with PDMS to form efficient wearable sensors is graphene quantum dots (GQDs). The variation in the graphene form has led to the formation of highly robust prototypes due to the strong interfacial bonding with the PDMS substrates. Some of the attributes of these materials are ultra-small size, non-toxicity, biocompatibility, and excellent chemical and thermal stability [145]. One of the interesting works is shown in [126], where GQDs/PDMS-based wearable sensors have been developed for quantifying the ovarian cancer biomarker CA-125. The sensors used chemiluminescence resonance energy transfer process to record the antigen from the samples that consisted of an equal ratio of human blood plasma and phosphate buffered saline (PBS) buffer. The sensors formed on amino-modified glass chips were salinized with 3-aminopropyl-trimethoxysilane layer and GQDs. The electrostatic attraction was used to immobilize the GQDs confined in the PDMS stencils. The GQDs-attached APTMS layer was capable of capturing the antibody that was specific to the CA-125 antigen. Covalent bonds were formed between the GQDs and antigen through the conjugation of amides. Bovine serum albumin was used during the reactions to place the unreacted sites on the glass templates. The increased amount of captured antigens simultaneously increased the concentration of antibodies bound near the GQDs. This leads to an increase in the amount of energy transfer, thus leading to chemiluminescence quenching. The sensors displayed a linear range from 0.1 U/mL to 600 U/mL and a high coefficient of determination (R^2^) of 99.6%. The prototypes have a LOD of 0.05 U/mL and an SNR of 3. Ye et al. [146] showcased the development of electrochemical biosensors for rapid and sensitive detection of bacteria response of antibiotics using nanoporous membranes. The prototypes were developed by integrating the PDMS chamber using bio-functionalized nanoporous alumina membrane. The chambers were divided into upper and lower parts, where two platinum electrodes were inserted in both the chambers to operate as working and reference electrodes, respectively. The GQDs present in the sensors were modified using anti-salmonella antibodies and were immobilized on the salinized nanoporous alumina membranes for the detection purposes. The response of the sensors was measured in terms of impedance during the capturing of the bacteria by antibodies on the membrane. There was change in the shape and size of bacteria during the functionalization of antibiotics on the Salmonella bacteria for a few minutes. These nanoporous membranes were able to detect the target bacteria within 30 min. The operating range of the sensors was between 1 nM and 100 µM. With R^2^ values of 0.9423 and 0.9348, the sensors showed a LOD of 1 pM and 40 pM for the antibodies enrofloxacin and ampicillin, respectively. The change in impedance was around from 1.7% to 10% and from 1.5% to 8.3% for the enrofloxacin and ampicillin antibodies, respectively.

**Figure 6:**
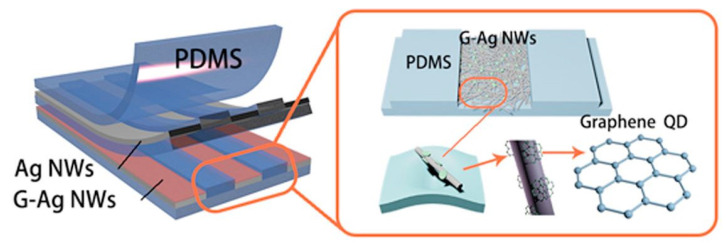
Schematic diagram of the structure of GQDs/PDMS-based e-skin [147]. Reproduced from Xu, Z., Wu, C., Li, F., Chen, W., Guo, T. and Kim, T.W., 2018. “Triboelectric electronic-skin based on graphene quantum dots for application in self-powered, smart, artificial fingers”. *Nano Energy*, *49*, pp. 274–282.

Regarding the strain-sensing applications of these GQDs/PDMS-based wearable sensors, Zhang et al. depicted the GQDs/PDMS-based strain sensors [134]. While the substrates of the prototypes were formed using PDMS, the sensing area was formed by mixing CNTs and GQDs at defined ratios. After curing the PDMS substrates, they were cut into rectangular shapes of specific dimensions. Copper wires were then attached to the substrates to use as bonding pads. Finally, the mixture of CNTs/GQDs was placed on the sensing area and attached to the prototypes using uncured PDMS. The average diameter of the chosen GQDs was between 15 and 20 nm. The CNTs and GQDs were mixed by adding the two aqueous solutions at five different mass ratios. Then, the mixture was dried on the centrifuged tubes at a temperature of 80 °C. The presence of both the nanomaterials in the sensing area increased the effective conductive pathways of the strain sensors. The dynamic change in the conductive pathways led to a tunneling mechanism in the composites, thus increasing the G.F. range of these sensors. The response of the prototypes was determined in terms of relative resistance with respect to the applied strain. The G.F. of these sensors had a range from 0 to 841.42. Another example related to this category can be shown in Xu et al. [147]. Triboelectric electronic skins were fabricated and employed as self-powered, smart, and artificial fingers. Some of the attributes of these sensors include light weight, high transparency, and high mechanical stretchability. Micro-gaps induced certain characteristics such as pressing, stretching, folding, and twisting. Figure 6 [147] shows the schematic diagram of the basic structure of these electronic skins (e-skins). After the patterning of the PDMS films was done, spin-coating of a photoresist film having a thickness of 5 µm was done on glass substrates. Then, the photolithography process was used to form uniform line arrays having both linewidth and a gap of 100 µm. The patterned photoresist was used as a mold to subsequently form the patterned PDMS films.

Finally, free-standing PDMS film was formed by annealing the film at 120 °C for an hour. Then, silver nanowires were deposited on the oxygen plasma-treated PDMS film to form the bottom electrode array. Then, the blade coating of the GQDs was done on the surface of the silver nanowires film, followed by annealing the samples at a temperature of 90 °C for 20 min. The step included covering the samples with another layer of free-standing PDMS films coated with a GQDs-patterned silver nanowire network. The second layer acted as the top electrode array for these triboelectric sensors. The application of pressure of 30 N obtained an output short-circuit current density range between 10 and 20 mA/cm^2^.

## 3. Challenges of the Existing Sensors

Although a lot of work has been done on developing graphene/PDMS-based sensors and using them for wearable sensing applications, there are still some issues that need to be addressed in the current scenarios. From the fabrication point of view, the formation of homogenous aqueous solutions with graphene or its by-products still needs to be addressed. Although certain solvents—such as methyl ethyl ketone (MEK), N, N-dimethyl acetamide (DMA), and N, N-dimethyl formamide (DMF)—can form homogenous solutions [148], the presence of these solvents disrupts the mechanical integrity of graphene overall electrical conductivity of the electrodes. The absence of the bandgap in graphene decreases the control on the following current. This can cause a problem, especially when the graphene electrodes are used as switches to control the current follow. Although the formation of nanocomposites might be helpful for certain wearable sensors, certain limitations—such as component stability, structural integrity, uncertain cytotoxicity, and mechanical instability—decrease the performance of the prototypes [149]. Due to the hydrophobic nature of PDMS, it is difficult to deposit additional layers on them. Thus, inducing selectivity on PDMS-based sensors is still an issue for the glucose-based electrochemical sensors. The highly deformable nature of PDMS can interfere with the electrochemical sensing applications of these sensors. The change in current due to the respective ions can be erroneous because of the bending of the sensors. The agglomeration of different graphene nanomaterials in the PDMS matrix is another area to be worked upon. Due to the difference in the physiomechanical structure of the aforementioned graphene nanomaterials, it is difficult to standardize the percolation threshold of these nanofillers.

Integrating these flexible wearable sensors with wireless communication protocols should be encouraged to execute the experiments in real-time situations rather than controlled environments. The consideration of flexible printed circuit boards (FPCBs) [150,151] should include formation of fully flexible smart-sensing systems. In addition to the integration of certain communication protocols—such as radio-frequency identification tags (RFID) [152,153] and medical internet of things (IoT) protocols [154,155]—to these wearable sensors, the data traffic should be reduced by big data and the simultaneous optimization of the sensor monitoring network [156]. The need for multifunctional wearable sensors is another matter to address in the current era. Apart from the fact that they can provide multiple measurements [157], they can also increase the compactness and structural integrity of the system. The reduction in the number of sensors while maintaining the multi-parametric detection capability can help in the subsequent reduction in the consumption of input power and convenient processing of sensed signals. From an application point of view, wearable sensors can be used to form cartouche systems for using them as point-of-care (POC) devices. The use of these wearable sensors should be increased with elderly people to determine the presence of any abnormality and increase life expectancy. A transparency and privacy of the collected data should be kept between the patients and monitoring unit to ensure the trust between the two parties under consideration. Along with the rectification of these above-mentioned problems, further work should be done to devise novel graphene/PDMS-based wearable sensor experimentation on several kinds of biomedical, environmental, and industrial applications.

## 4. Conclusions

The paper illustrates the work done on graphene/PDMS-based sensors for wearable sensing applications. These sensors have included graphene and PDMS as processing materials due to their biocompatibility, biodegradability, and excellent mechanical characteristics. In addition to that, graphene also has excellent electrical properties that also assisted the prototypes in achieving enhanced performances. In addition to graphene and PDMS, the adjoining of other biocompatible nanomaterials and polymers forms the resultant multilayered and multifunctional wearable sensors. The wearable sensors have been used as electrochemical and strain-sensing prototypes to detect the physiological signals and parameters, respectively, of the human body. When used for rehabilitation purposes, these sensors can determine and improve the cognitive behavior of human beings. The embedding of these wearable prototypes with various communication protocols and proper scrutiny of the sensed data would help determine the critical data from the big data. The real-time application of these sensors, if done, can assist in dealing with physiological and psychological problems of a wide section of society.

## Figures and Tables

**Table 1:** Comparative study on the quality of graphene and other nanomaterials used to develop glucose-based electrochemical sensors.

Materials Used	Fabrication Method	Linear Range	Sensitivity	Limit of Detection	Ref.
GN, PDMS, Gold NPs	Micromolding	0–162 mg/dL	10 mA/cm^2^	1.44 mg/dL	[123]
rGO, PDMS, SiO_2_, Nafion	Electrostatic interaction, in situ reduction	0.1–9 mM	60.8 µA·mM^−1^·cm^−2^	3.7 µM	[124]
GNP, CeO_2_, PDMS, PANI	Magnetic stirring	5–100 µM	29.35 ± 1.4 μA·μM^−1^	0.14 µM	[125]
GQD, PDMS	Salination	0.1–600 U/mL	-	0.05 U/mL	[126]
Co_3_N NWs, Titanium	Autoclave treatment	0.1 μM–25 mM	3325.6 μA·mM^−1^·cm^−2^	50 nM	[127]
Co-based MOF, Ag@ZIF-67, Glassy carbon electrode	Sequential deposition-reduction	2–1000 μM	0.379 μA·μM^−1^·cm^−2^	0.66 μM	[128]
Gold, palladium naowires	Electrochemical nanowire assembly	10^−6^–10^−3^ M	18 μA·M^−1^	3 × 10^−7^ M	[129]
Gold-Nickel oxide nanowires	Plasmon method	0.005–15 mM	4.061 mA·cm^−2^·mM^−1^	0.005 mM	[130]

GN: Graphene nanopowder; PDMS: Polydimethylsiloxane; GNP: Graphene nanoplatelets; CeO_2_: Cerium oxide; PANI: Polyaniline; Co_3_N: Cobalt nitride nanowires; SiO_2_: Silicon dioxide; Co-based MOF: Cobalt-based metal-organic framework.

**Table 2:** Comparative study on the quality of graphene and other nanomaterials-based strain sensors.

Materials Used	Fabrication Method	Linear Range	Gauge Factor	Limit of Detection	Ref.
GN, PDMS, Ti_3_C_2_T_x_	Magnetic stirring	0–74.1%	190.8 (0–56%), 1148.2 (52.6–74.1%)	−0.025% (lower)	[131]
rGO fiber fabrics, PDMS	Vacuum filtration	0.24–70%	1668.48	1.17 Pa (higher)	[132]
GNP, PDMS	Coating	0–65%	62.5	65% (higher)	[133]
GQD, CNTs, PDMS, Copper wires	Drop casting	7%	841.2	19% (higher)	[134]
Ag NWs, Dragon skin	Embed-and-transfer	0–150%	81 (>130% strain)	150% (higher)	[135]
Au NWs, PU nanofibers	Electrospinning	0–70%	12 (5% strain)–2379 (70% strain)	70% (higher)	[136]
EGaIn, Ecoflex	Electroless plating	-	21–25	320% (higher)	[137]
Ag NWs, PDMS	Vacuum filtration and trasfer	0–10%	>20	35% (higher)	[138]

GN: Graphene nanopowder; PDMS: Polydimethylsiloxane; Ti_3_C_2_T_x_: Titanium carbide; GNP: Graphene platelets; rGO fiber: Reduced graphene oxide fiber; GQD: Graphene quantum dots; Ag NWs: Silver nanowires; PU: Polyurethane; EGAIn: Eutectic gallium indium; Ag NWs: Silver nanowires.

## Data Availability

Not applicable.
